# Cardiovascular Subphenotypes in ARDS: Diagnostic and Therapeutic Implications and Overlap with Other ARDS Subphenotypes

**DOI:** 10.3390/jcm12113695

**Published:** 2023-05-26

**Authors:** Minesh Chotalia, Jaimin M. Patel, Mansoor N. Bangash, Dhruv Parekh

**Affiliations:** 1 Birmingham Acute Care Research Group, University of Birmingham, Birmingham B15 2SQ, UK; 2 Department of Anaesthetics and Critical Care, Queen Elizabeth Hospital Birmingham, Birmingham B15 2GW, UK

**Keywords:** acute respiratory distress syndrome, latent class analysis, right ventricular dysfunction, right ventricular failure, subphenotypes, transthoracic echocardiography

## Abstract

Acute respiratory distress syndrome (ARDS) is a highly heterogeneous clinical condition. Shock is a poor prognostic sign in ARDS, and heterogeneity in its pathophysiology may be a barrier to its effective treatment. Although right ventricular dysfunction is commonly implicated, there is no consensus definition for its diagnosis, and left ventricular function is neglected. There is a need to identify the homogenous subgroups within ARDS, that have a similar pathobiology, which can then be treated with targeted therapies. Haemodynamic clustering analyses in patients with ARDS have identified two subphenotypes of increasingly severe right ventricular injury, and a further subphenotype of hyperdynamic left ventricular function. In this review, we discuss how phenotyping the cardiovascular system in ARDS may align with haemodynamic pathophysiology, can aid in optimally defining right ventricular dysfunction and can identify tailored therapeutic targets for shock in ARDS. Additionally, clustering analyses of inflammatory, clinical and radiographic data describe other subphenotypes in ARDS. We detail the potential overlap between these and the cardiovascular phenotypes.

## 1. Introduction

Acute respiratory distress syndrome (ARDS) is a severe, life-threatening condition that is characterised by the presence of acute hypoxaemic respiratory failure and lung inflammation [1,2,3,4]. It is defined by a PaO_2_:FiO_2_ ratio of <300 mmHg and the presence of bilateral opacification when chest imaging (not fully explained by left ventricular failure) at a positive end-expiratory pressure (PEEP) of 5 cm H_2_O and occurring within 7 days of an insult (Berlin definition [5]). ARDS is common in patients admitted to the intensive care unit (ICU) (present in 10.6% of ICU admissions [6]) and, despite six decades of research, has a high attributable mortality (46% in severe ARDS [6]). The syndrome has a broad range of triggers that are both direct (pulmonary) and indirect (extrapulmonary) and include pneumonia, aspiration, sepsis, trauma, blood transfusion, smoke inhalation, drowning and pancreatitis [4]. It has a complex pathophysiology of dysregulated inflammatory, coagulation and injury pathways, potentially exacerbated by mechanical ventilation, that cause alveolar epithelial and/or pulmonary endothelial damage [3].

Shock is frequent in ARDS and is associated with high morbidity and mortality [7,8]. Right ventricular dysfunction (RVD) is commonly implicated as the cause of shock [9,10,11,12]. This may be because at least mild–moderate acute pulmonary arterial hypertension (PAH) develops in most, precipitated by numerous factors: direct pulmonary endothelial injury/inflammation, micro-thrombi, hypoxaemia, hypercarbia, acidosis and/or raised intrathoracic pressure from positive pressure ventilation [13]. The thin-walled right ventricle, adapted to ejecting blood into a low-resistance, highly compliant pulmonary circulation, struggles to expel blood against an increased pulmonary afterload, precipitating venous and organ congestion along with reduced right ventricular (RV) and, thereby, left ventricular (LV) cardiac output [14]. RVD is modifiable with the application of a prone position [15], lung-protective ventilation [16] and inodilator therapies [17]. The prevention and treatment of RVD is, therefore, an important therapeutic avenue.

## 2. Literature Search

A PubMed search was conducted, and relevant articles addressing cardiovascular function in ARDS and subphenotypes in ARDS from January 2000 to October 2022 were reviewed for inclusion. The following search terms were used: “acute cor pulmonale” [All Fields] OR “right ventricular dysfunction” [All Fields] OR “subphenotype” [All Fields] OR “phenotype” [All Fields] OR “subtype” [All Fields] OR “cardiovascular” [All Fields] OR “shock” [All Fields] AND “ARDS” [Title/Abstract] OR “acute respiratory distress syndrome” [Title/Abstract] OR “Acute Lung Injury” [Title/Abstract] OR “ALI” [Title/Abstract] OR “respiratory distress syndrome” [MeSH Terms].

## 3. The Problem

It has never been in doubt that ARDS is a syndrome encompassing many different disease states [18,19,20]. A broad, syndromic definition made ARDS common, easier to identify and more generalizable to routine clinical practice, which aided in recognising patients for potentially beneficial treatments (lung protective/prone ventilation) and later facilitated recruitment into clinical trials/observational studies [21].

However, a broad ARDS definition necessarily results in heterogeneity in its triggers (pulmonary vs. extra-pulmonary), severity, distribution of injury (focal vs. diffuse lung injury), pathophysiology (epithelial vs. endothelial injury; alveolar vs. systemic inflammation), time frame (early vs. late in ICU admission) and duration (rapidly resolving vs. prolonged) [18]. It is increasingly apparent that this same heterogeneity is a barrier to producing targeted and, therefore, effective therapies, evidenced by over 150 randomised controlled trials of pharmacological therapies in ARDS that failed to demonstrate significant patient benefit [22].

Understandably, the cardiovascular manifestations of ARDS may also be diverse. Whilst RVD is commonly implicated in ARDS-related shock, this itself is problematic because RVD has no consensus definition [23]. A recent meta-analysis identified that, in 13 studies assessing RVD in 1861 patients with ARDS, nine definitions were employed, using three different modalities [24]. The American and British societies of echocardiography define RVD as “RV systolic impairment” [25,26,27], and an ESICM statement used “RV dilation with evidence of systemic congestion” [28], whilst a state-of-the-art paper used “an inability of the RV to meet blood flow demands without excessive use of the Frank-Starling mechanism” [29]. Acute cor pulmonale (ACP) is most frequently used in the literature to define RVD [11,12,30,31,32,33,34]: RV:LV end-diastolic area (RV:LVEDA) > 0.6 and septal dyskinesia. In the largest prospective study in moderate–severe ARDS to date (*n* = 752), ACP was not associated with mortality. Although severe ACP (RV:LVEDA > 1) was, its incidence was low (7%) in this [11] and other studies [35]. In contrast, ACP was associated with mortality in COVID-19 ARDS (C-ARDS) in a large retrospective multicentre study of 677 patients [33]. In a similar manner, other descriptions of RVD were also inconsistently associated with mortality [24], including newer measurements such as RV free wall longitudinal strain [36,37].

The limitations of the current RVD definitions include the following:

1. Utilising a limited number of variables that can be difficult to accurately measure given the complexity in RV geometry [38];
2. Using arbitrary, unvalidated cut-off values for these variables in a binary manner, which means that patients at the border of this cut-off may frequently change groups;
3. Neglecting the effect of RVD on LV function, cardiac output, despite the RV being connected in series to the LV, and ventricular interdependence, meaning that RVD can negatively impact LV filling [39];
4. Overlooking the predominant LV pathology that may precipitate shock in ARDS [40] by solely focusing on the right ventricle; this surprising oversight occurs despite ARDS sharing notable overlap with sepsis, a condition where septic cardiomyopathy [41] and hyperdynamic LV ejection fraction (HDLVEF; [42,43]) are frequent and associated with poor outcomes.

An RVD definition, which associates with mortality is prevalent, integrates global cardiovascular (CV) function and aligns with known pathophysiology is, therefore, needed [44].

## 4. Clustering Analysis: A Possible Solution

Clustering analyses may be used in ARDS to group patients with similar patterns of observed clinical, biological, radiological or outcome variables together, generating homogenous subgroups with comparable characteristics/pathophysiology (subphenotypes) from heterogeneous parent populations [45,46,47,48,49,50,51]. The expectation is that these similarities extend to treatment characteristics, with the patients within a subphenotype all favourably responding to a particular intervention—i.e., possessing a “treatable trait”.

This approach has many potential advantages in characterising the CV system:

1. It is data driven and, therefore, unbiased;
2. It can incorporate multiple haemodynamic variables that assess global cardiovascular function;
3. It identifies the states of CV dysfunction that commonly occur and are, therefore, more likely to describe genuine pathophysiology [52];
4. Trials of therapies in these subphenotypes will benefit from predictive and prognostic enrichment, by using treatments targeting the aberrant pathophysiological process in a subgroup with a high-risk of mortality [18].

Our group previously performed a latent class analysis (LCA) of transthoracic echocardiography (TTE) and clinical haemodynamic variables in 305 C-ARDS [50] and 801 non-C-ARDS patients [51]. The TTE and clinical parameters used in both studies included RV:LVEDA to denote RV size, RV fractional area change and tricuspid annular plane systolic function (TAPSE) to denote RV systolic function, inferior vena cava diameter, LV ejection fraction, cardiac index, heart rate, central venous pressure and vasopressor dose. Patients with pre-existing cardiac abnormalities on TTE were excluded in an effort to characterise the haemodynamic changes secondary to the pathology itself (ARDS) and not those already existing in patients. Both studies identified two subphenotypes of increasingly severe right ventricular injury in ARDS that were conserved across both conditions. The first RV subphenotype had a mortality rate of 42% in C-ARDS and 40% in ARDS but was not independently associated with mortality itself and was characterised by a state of RV dilation with preserved RV/LV systolic function and cardiac output. The second RV subphenotype was described by RV dilation with RV systolic impairment, impaired LV filling and cardiac output and had a much higher mortality rate (73% in C-ARDS and 78% in ARDS [50,51]) and an independent association with mortality (OR 6.9 (4.0–11.8) [51]). A third subphenotype, only found in the ARDS population, mirrored HDLVEF cohorts in sepsis and was characterised by high cardiac output, low systemic vascular resistance (SVR) and a high mortality rate (59% died; OR 2.5 (1.6–3.7) [49]). Both study populations also included “normal” cardiovascular subphenotypes with largely normal TTE/CV parameters and low mortality rates [50,51]. In Section 5, we discuss the pathophysiological, diagnostic and therapeutic implications of the abnormal subphenotypes.

## 5. Pathophysiological Implications

**Figure 1:**
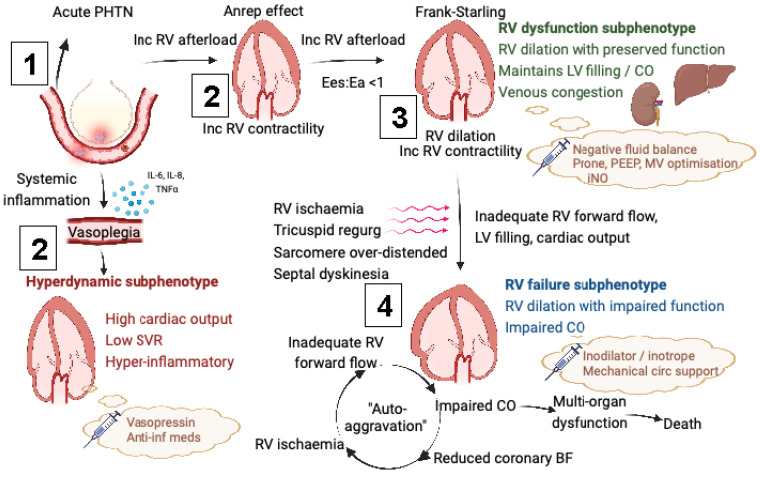
Pathophysiology of cardiovascular subphenotypes in ARDS. Numbers indicate step-wise transition from baseline (1) to deranged physiology (2,3,4). PHTN = pulmonary hypertension; RV = right ventricular; Ees = end-systolic elastance; Ea = arterial elastance; LV = left ventricular; CO = cardiac output; PEEP = positive end-expiratory pressure; MV = mechanical ventilation; iNO = inhaled nitric oxide; regurg = regurgitation; circ = circulation; BF = blood flow; anti-inf = anti-inflammatory; SVR = systemic vascular resistance; IL = interleukin; TNF = tumour necrosis factor. Created with Biorender.com (accessed on 27 November 2022).

The two identified RV subphenotypes align with previously described RVD pathophysiology (Figure 1). The first could be explained by an increased RV afterload exhausting homeometric adaptation (Anrep effect) and inducing right ventricular:pulmonary arterial (RV:PA) uncoupling (Ees:Ea < 1) [53]. This in turn precipitates RV dilation, cardiomyocyte stretch and, via the Frank-Starling mechanism (heterometric adaptation) [54], an increase in RV contractility, which critically restores RV:PA coupling and maintains RV systolic function, LV filling and cardiac output, preserving forward flow, albeit at the expense of venous congestion [55]. This may be why this RV subphenotype did not independently associate with mortality but had a higher incidence of renal replacement therapy [56,57].

Decompensation from this state of adapted RV dilation may be precipitated by four potential mechanisms, occurring together or in isolation, which result in inadequate LV preload and cardiac output [14]:

1. Raised RV end-diastolic pressure precipitating subendocardial ischaemia by only allowing coronary blood flow in systole [58];
2. Marked septal dyskinesia impairing LV filling and output (ventricular interdependence) [59];
3. Excessive RV dilation decreasing RV stroke volume by
  - Stretching the tricuspid annulus precipitating tricuspid regurgitation [60];
  - Lengthening sarcomeres above their optimal interactive capacity.

Inadequate cardiac output is then the critical step in triggering the vicious cycle of auto-aggravation [14]; whereby, low cardiac output further reduces coronary perfusion, aggravating RV ischaemia and worsening RV contractility, whilst also reducing end-organ perfusion, which triggers multi-organ dysfunction and death.

## 6. Diagnostic Implications for RVD

This has potential implications for defining RVD. Rather than one category of RVD, we propose that at least two states of RV injury may be present in ARDS and require delineation due to differing clinical and outcome characteristics: one is compensated RV dilation (which may have a minimal, independent effect on mortality), and the other is RV failure (which may strongly associate with mortality [50,51]). The temporal relationship between the two is currently unclear. RV dilation is common in both forms of RVD (although more likely severe in the latter), but we suggest that impaired RV systolic function and cardiac output are the critical parameters for differentiating the RV failure subtype.

An LCA cannot be used at the bedside to identify patients in specific RV subphenotypes. However, parsimonious logistic regression techniques can identify models that use only three variables to describe which LCA-derived subphenotype a patient belongs to with high sensitivity and specificity. These can then be used at the bedside to prospectively identify patients. The compensated RV dilation subphenotype was described with high sensitivity and specificity by using three-variable models with RV:LVEDA > 0.6, RVFAC > 0.25 and TAPSE > 16, whereas the RV failure subphenotype was described by RV:LVEDA > 0.6, RVFAC < 0.35 and cardiac index < 4. Therefore, it is the presence of RV systolic dysfunction and low cardiac index that appears to differentiate the RV failure subphenotype from the compensated RV dilation subtype.

Furthermore, RV dilation and systolic impairment were used to describe a high-risk mortality RV phenotype in ARDS [51], C-ARDS [61] and sepsis [62] cohorts, but, they were unable to reliably differentiate the two LCA-derived RV subphenotypes [50,51]. This may be because they do not hold information about the adequacy of cardiac output, which may be the key to preventing coronary insufficiency and end-organ hypoperfusion and can result in RV ischaemia as well as multi-organ dysfunction [14]. Indeed, the key to differentiating the two RV subphenotypes using three variable models was the normal cardiac index and RVFAC in the low-risk of mortality “compensated RV dilation” subphenotype, with these parameters being abnormal in the high-risk of mortality “RV failure” subtype. However, inaccuracies in the measurement of TTE-derived cardiac output may also impact its utility as a diagnostic marker [63].

Based on the current evidence, we propose RV dilation with preserved systolic function and/or cardiac output and RV dilation with impaired systolic function and cardiac output as two states of RV injury in ARDS. The first RVD subtype aligns with the state-of-the-art definition of RVD: “an inability of the RV to meet blood flow demands without excessive use of the Frank-Starling mechanism” [29], whereas the second is defined by the RV being “unable to meet blood flow demands despite excessive use of the Frank-Starling mechanism”.

This work requires prospective validation in multi-centre studies. Assessment of RV subphenotype stability across ICU stays and the relationship to therapies that may modify cardiovascular function is also required. Newer parameters such as RV:PA coupling [64], RV free wall longitudinal strain [36,37] and RV diastolic dysfunction [65] as well as markers of venous congestion (hepatic and renal venous doppler assessment [55,66]) should also be incorporated, as increased data dimensionality may change the number or characteristics of the subphenotypes.

## 7. Therapeutic Implications

Treatment of RV dysfunction in ARDS was discussed in detail in many prior reviews [28,29,30,39,53].

Briefly, the general principles include the following:

1. Reducing RV afterload, either by
  - Correcting parameters that contribute to PAH, e.g., driving pressure, hypercarbia, acidosis and hypoxia with RV-protective ventilation strategies, e.g., prone position ventilation [67];
  - Using pulmonary artery vasodilators, e.g., inhaled nitric oxide [68];
2. Increasing RV forward flow using inotrope medications [17] or mechanical circulatory support [69];
3. Combatting the deleterious effects of venous congestion by achieving negative fluid balances with either diuretics or renal replacement therapy [70].

To the best of our knowledge, none of these treatments have been selectively applied to ARDS patients with RVD in RCTs to assess their benefit. Application of prone position ventilation has a mortality benefit in patients with severe ARDS [71]. Whether it can improve outcomes for patients with mild–moderate ARDS with RVD warrants investigation [72], particularly given that it augments RV function [15] and brings about greater changes in blood gas parameters in patients with RVD than in those without [50,61].

Clearly, these treatment modalities lie on a spectrum of invasiveness/risk and should be differentially applied to RV injury states by addressing the underlying pathophysiology. For example, in cohorts with compensated RV dilation, reducing RV afterload and venous congestion would be expected to mitigate progression to decompensated RV failure—this warrants investigation. On the other hand, in patients where RV systolic function and cardiac output are compromised, so prognosis is much poorer, the above therapies may be inadequate to reverse the already dire clinical state. Instead, trials of inotrope/inodilator therapies or mechanical circulatory support could benefit from predictive as well as prognostic enrichment. Indeed, the presence of RV failure was found to better identify patients with survival benefit from veno-venous extracorporeal membrane oxygenation in ARDS [73].

## 8. Left Ventricular Function in ARDS

Clustering analysis also identified a hyperdynamic left ventricular ejection fraction (HDLVEF), high-cardiac-output subphenotype in ARDS but not in C-ARDS [50,51]. This state is synonymous with low systemic vascular resistance (or vasoplegia), high inflammatory markers, renal dysfunction and high mortality [51]. Identifying this subgroup in ARDS is unsurprising, given the notable overlap with sepsis [8,74], a syndrome where vasoplegia [75] and hyperdynamic cardiac output states [43] are consistently identified. HDLVEF cardiac subgroups are also associated with poor outcome in unselected critically ill populations [42]. The cause(s) of this hyperdynamic state in ARDS are unknown, but, theoretically, high cardiac output may be required to maintain end-organ perfusion in the setting of profound vasodilation [75] and could, therefore, be interpreted as a compensatory adaptation. In septic populations, vasoplegia was independently associated with the development of this subphenotype [43]. Other potential causes include a hyperadrenergic state, hypovolaemia and diastolic dysfunction [43]. Indeed, diastolic dysfunction was identified in ARDS and is associated with increased mortality but not with HDLVEF states [40].

Diagnostically, this suggests that a solely RV-centric view of shock in ARDS may be too simplistic and may neglect patients with relatively preserved RV function but a high likelihood of developing refractory shock and death. Future studies should prospectively evaluate the incidence, patient characteristics and associated mortality of this subphenotype in ARDS populations to better identify potential therapeutic options. If vasoplegia is a predominant cause, vasopressor agents devoid of inotropic action such as vasopressin could counteract vasodilation without concomitantly increasing cardiac output and myocardial oxygen demand. Indeed, in a large, multi-centre randomised controlled trial, vasopressin reduced the need for renal replacement therapy compared to noradrenaline in patients with septic shock [76]. Whether selective beta blockade may be of benefit in HDLVEF is also unclear [77]. If hyperadrenergism or diastolic dysfunction dominate as the cause, then benefit could be expected. However, recent evidence suggests harm could also result from this treatment modality, potentially signifying that either the high-cardiac-output state is compensatory in nature or hyperadrenergism is not the dominant driver of HDLVEF [78].

## 9. Limitations

Retrospective studies are limited by selection bias: the decision to perform a TTE was in those with elevated troponin in the C-ARDS cohort [50] at the discretion of the treating intensivist in the non-C-ARDS cohort [51], and this may have selected a cohort at higher risk of having LV/RV dysfunction. Therefore, not all patients with ARDS received a TTE, so whether these subphenotypes exist in all ARDS patients warrants prospective investigation. Nonetheless, patients that did not receive a TTE were unlikely to have grossly abnormal cardiovascular function, and, hence, their exclusion was unlikely to markedly influence subphenotype derivation. Patients with pre-existing LV/RV dilation or systolic dysfunction were excluded from these studies; however, patients with undiagnosed but chronic cardiac dysfunction may have been included. The studies also require external validation in multi-centre cohorts, as patient demographics and the causes of and treatments for ARDS may differ between centres, although identifying the two RV subphenotypes in both C-ARDS and non-C-ARDS is promising. TTE is non-invasive and inexpensive and is increasingly being utilised to assess shock states in critically ill patients; hence, we believe that characterising cardiovascular function using TTE-derived parameters is appropriate. Nonetheless, inaccuracies in the measurement of RV size and function and cardiac output [31] may be an important limitation to this work. This was reduced by the following:

(i) Employing a clustering approach that incorporates multiple parameters to delineate subphenotype class, rather than focusing on one or two parameters per the existing definitions of RVD;
(ii) Excluding patients with inadequate TTE views from the study;
(iii) Ensuring TTEs were performed by practitioners with advanced accreditation only.

## 10. Potential Overlap with Other ARDS Subphenotypes

**Figure 2:**
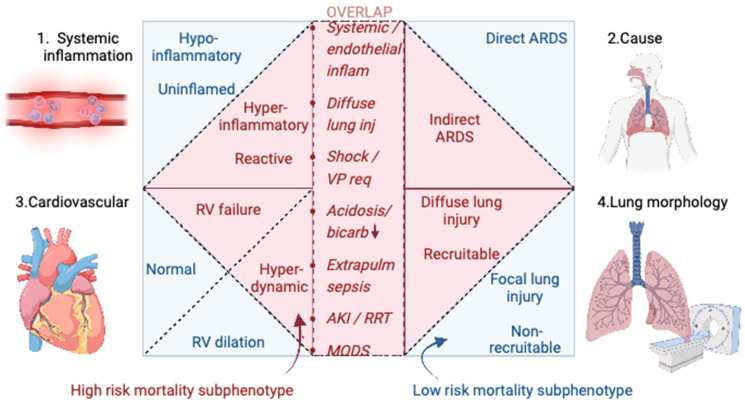
Potential overlap between ARDS subphenotypes/subgroups. Four different domains of subphenotypes/subgroups are identified (1. systemic inflammation, 2. cause or risk factor for ARDS, 3. cardiovascular and 4. lung morphology/respiratory). The high risk of mortality subphenotypes from these domains, identified in red shading, overlap in some aspects: systemic/endothelial inflammation (inflam), diffuse lung injury (inj), shock/vasopressor requirement (VP req), metabolic acidosis/low bicarbonate (bicarb) levels, extrapulmonary (extrapulm) sepsis, acute kidney injury (AKI) or renal replacement therapy (RRT) and multiorgan dysfunction syndrome (MODS). Created with Biorender.com (accessed on 28 November 2022).

The heterogeneity of ARDS populations, unsurprisingly, extends to other organ systems/biological pathways beyond the cardiovascular system. Efforts to parse inflammatory [46,47,48,49,79,80,81,82], respiratory [83] and lung morphological data [84,85] from ARDS cohorts into homogeneous subphenotypes are extensive and predate the work performed on cardiovascular subphenotyping. Here, we describe the other subphenotypes/subgroups present in the ARDS literature and consider their similarities to and differences from the cardiovascular clusters discussed above (Figure 2 and Table 1).

The subphenotypes generated using a latent class analysis of the plasma biomarkers/cytokines and clinical data from >3000 patients from five multi-centre randomised controlled trials [46,47,48,49], two observational cohorts [79,80] and pre-clinical models [81] of ARDS are the most extensively validated. Two subphenotypes are consistently described and labelled in terms of an inflammation-centric model: hyper- and hypo-inflammatory ARDS. The hyper-inflammatory subgroup is consistently associated with a higher mortality rate and is characterised by a greater incidence of extra-pulmonary sepsis/indirect ARDS, biologically by higher inflammatory plasma cytokine concentrations (e.g., IL-6, IL-8, sTNFR1, Ang-2 and sRAGE) and clinically in large part by features of shock (vasopressor use, tachycardia and lower systolic blood pressure), metabolic acidosis and renal, liver and coagulative dysfunction [46,47,48,49]. These inflammatory subphenotypes are also consistently aligned with characteristic leukocyte transcriptomic responses and signalling pathways [82] and may be identifiable using readily available clinical data alone [80]. The potential importance of these inflammatory subphenotypes was shown in unspecified post hoc subgroup analyses, where lower mortality was observed in the hyper-inflammatory subphenotype when receiving high PEEP [46], liberal fluid [48] and simvastatin [49] therapies.

Clearly, the constellation of hyper-inflammation, extra-pulmonary and diffuse lung injury, shock, tachycardia and renal/liver/coagulative dysfunction shares many features with the HDLVEF subphenotype found in sepsis and ARDS populations [42,43,51]. The haemodynamic features of this subphenotype suggest that a more liberal fluid strategy may be of benefit to this cohort, as was also the case in patients with hyper-inflammatory ARDS. However, some of these features (shock, renal/liver dysfunction, and high mortality) are also notably shared with the RV failure subphenotype [51]. This subtype may overlap with hyper-inflammatory ARDS as heightened immune mediated processes (NETosis-related immunothrombosis [86], monocyte recruitment and inflammasome activation [87]) may precipitate RVD in a similar manner to septic cardiomyopathy. However, RVD was, previously, more synonymous with direct lung injury [49], and pneumonia is more of a risk factor in its development [11] than in indirect and extra-pulmonary ARDS. Nonetheless, a single hyper-inflammatory subphenotype may be parsed into two cardiovascular subphenotypes as a result of the organ-specific information included in the cardiovascular LCA. Aside from CVP, there were no meaningful differences in haemodynamic variables (pulmonary artery occlusion pressure and cardiac output) between hyper- and hypo-inflammatory subgroups [48]. This may be because the hyper-inflammatory ARDS subgroup has a mixture of patients from the HDLVEF and RV failure CV subphenotypes. Their degree of overlap warrants a prospective investigation.

These features of hyper-inflammation also partially overlap with other ARDS subgroups: indirect ARDS [88] and diffuse ARDS [84,85]. Indirect ARDS (where the ARDS precipitant is extra-pulmonary, e.g., sepsis and pancreatitis) correlates with greater endothelial injury, vascular leak, interstitial oedema and diffuse ground glass opacification, with a relatively preserved alveolar space, and, as a result, may gain greater benefit from increased PEEP and lung recruitment strategies [89].

**Table 1:** Demographic, clinical, outcome and treatment-response characteristics of ARDS subphenotypes/subgroups.

Subphenotype Classification:	Systemic Inflammation [49,82]		Cause of Lung Injury [88,89]		Lung Pathophysiology [83]		Lung Morphology [84,85]		Cardiovascular [50,51]		
	Hyper-inf/Reactive	Hypo-inf/Uninflamed	Indirect	Direct	Recruitable	Non-Recruitable	Diffuse “Non-Focal”	Focal	RV Failure	HDLVEF	Normal
Prevalence	35%	65%	27%	73%	55%	45%	50%	50% *	13%	21%	43%
Characteristics											
Respiratory											
P/F ratio	121	132	136	128	144	193	240	217	188	210	210
Direct lung injury (%)	55%	85%	N/A	N/A	67%	43%	?	?	68%	42%	64%
Driving pressure/elastance/compliance	Lower Cstat, higher DP	Higher Cstat, Lower DP	Lower lung elastance	Higher lung elastance	Lung elastance 28 DP 17	Lung elastance 22 DP 12	Cstat 34 DP 12	Cstat 40 DP 11	Cdyn 30	Cdyn 32	Cdyn 31
Lung morphology on imaging	?	?	Diffuse, ground glass opacification	Focal, consoli-dation	Diffuse “inhomo-geneous”	Focal “homo-geneous”	Diffuse	Focal	Greater opacification score	Lower opacification score	Lower opacification score
Extra-organ dysfunction											
On vasopressor	81%	58%	49%	30%	58%	52%	No different	No different	83%	72%	55%
Scoring system	?	?	APACHE III 103	APACHE III 94	SAPS II 43	SAPS II 41	?	?	SOFA 10 (7–12)	SOFA 9 (7–12)	SOFA 6 (4–9)
pH/bicarbonate	Lower Lower	Higher Higher	No difference	No difference	Lower/higher	Higher/lower	Lower Lower	Higher Higher	Lower Lower	Lower Lower	Higher Higher
Creatinine, bilirubin, platelet	Higher Higher Lower	Lower Lower Higher	?	?	?	?	?	?	Higher Higher Normal	Higher Higher Lower	Lower Lower Higher
Systemic inflammatory (vascular) markers, e.g., IL-6, IL-8, TNF, RAGE Ang-2 and vWF	Higher	Lower	Higher	Lower	Higher sRAGE	Lower sRAGE	Higher	Lower	Higher WBC and PMN	Higher WBC, PMN and CRP	Lower WBC, PMN and CRP
Alveolar inflammatory markers	No difference	No difference	Lower	Higher	?	?	?	?	?	?	?
Mortality	40–60%	15–25%	90-day 35%	90-day 29%	ICU 52%	ICU 23%	90-day 28%	90-day 16%	90-day 78%	90-day 59%	90-day 19%
Treatment response											
Recruitment manoeuvre	?	?	Beneficial, including resp compliance	Harmful, decrease in resp compliance	Beneficial (including ventilated tissue, compliance and P/F)	No difference	Decrease in mortality (if correctly classified)	Increase in mortality	?	?	?
High PEEP	Beneficial	No difference	Beneficial (recruitment and dec elastance)	No difference	?	?	Decrease in mortality if correctly classified	Increase in mortality	?	?	?
Prone positioning	?	?	Beneficial (recruitment and dec elastance)	No difference	?	?	Harm if mis-classified	Mortality benefit if correctly classified	Greater improvement in blood gas parameters	?	Less improvement in blood gas parameters

Shaded column denotes high-mortality subphenotype/subgroup. Hyper-inf = hyperinflammatory subphenotype; Hypo-inf = hypo-inflammatory subphenotype; P/F = PaO_2_:FiO_2_ ratio; APACHE II = acute physiology and chronic health evaluation; SOFA = sequential organ failure assessment; SAPS = simplified acute physiology score; Cstat = static compliance; Cdyn = dynamic compliance; DP = driving pressure; IL = interleukin; sTNFR1 = soluble tumour necrosis factor receptor 1; sRAGE = soluble receptor for advance glucation end products; Ang-2 = angiopoietin 2; vWF = von Willebrand factor; PEEP = positive end expiratory pressure; WBC = white blood cell count; PMN = polymorphonuclear cell count; CRP = C-reactive protein. * This does not describe true prevalence, as patients were randomised 1:1 as part of randomised clinical trial. The existence of one subphenotype within a particular data domain (e.g., inflammatory) should not exclude the presence of subphenotypes in other domains (e.g., cardiovascular). In fact, a multitude of subphenotypes may exist as a result of the organ/patient specific data exposed to clustering analysis, reflecting the compartmentalisation of host responses—for example, systemic cytokine responses do not overlap well with bronchoalveolar lavage/alveoli inflammatory subphenotypes [90,91]. As such, patients may belong to multiple subphenotypes from different domains at once, with each denoting a potential treatable trait. Whilst inflammatory subphenotypes may aid in stratifying immunomodulatory therapies, cardiovascular or respiratory subphenotypes may help in personalising treatments for shock [51] and mechanical ventilation [83], respectively. Likewise, substantial overlap between subphenotypes may denote shared system response processes resulting in their manifestation and, if particularly robust, may tentatively suggest treatment modalities that extend to both. The finding that the inflammatory subphenotypes are conserved outside of ARDS, e.g., in mechanically ventilated patients without ARDS [92], acute kidney injury [93,94], pancreatitis [95] and sepsis survival [96] cohorts, suggest that these may be system response modes rather than ARDS (lung) response modes. Whether cardiovascular subphenotypes identified in ARDS extend to other critically ill populations, therefore, also warrants investigation.

Patients with diffuse lung injury (e.g., non-focal lung morphology) also exhibit greater lung recruitment with high PEEP and recruitment manoeuvres than those with focal ARDS [85]. An RCT of high PEEP/lung recruitment targeted to patients with diffuse ARDS (and low PEEP, prone ventilation administered to patients with focal ARDS) did not demonstrate a mortality benefit; however, this may be because 20% of patients were misclassified and, after exclusion of these patients, a mortality benefit was observed [84]. An LCA of radiological and respiratory/ventilatory data also described a “recruitable” subphenotype with greater lung injury, lower compliance, higher mortality and superior lung recruitment with open-lung ventilation strategies [83].

A unifying pathophysiological process for hyper-inflammatory, hyperdynamic and recruitable ARDS subphenotypes and diffuse and indirect ARDS subgroups could be heightened systemic endothelial inflammation precipitating vasoplegia, vascular leak and hyperdynamic cardiac function (Figure 2). This could bring about a more diffuse (non-focal) lung injury pattern with interstitial oedema and extra-alveolar collapse that responds well to high PEEP/recruitment. However, this may be an oversimplification, as there are also significant differences between these subphenotypes/subgroups: for example, the recruitable subphenotype had a greater proportion of patients with direct lung injury [83], and patients in the indirect ARDS subgroup had higher respiratory compliance [89]. Furthermore, this comparison is compounded by the different demographics of the study populations and non-standardised data collection/measurement, e.g., in terms of inflammatory markers.

The overlap between these subgroups and phenotypes warrants prospective validation incorporating cardiovascular, respiratory, lung morphological, transcriptomic and inflammatory variables. The pattern of subphenotypes is visible with organ specific subsets of parameters, so the subphenotypes might be expected to exist on an organ system basis too. However, the possibility of more hidden organ subphenotypes is not precluded, so a comprehensive multi-system array is needed.

## 11. Conclusions

Clustering analyses consistently identify two stages of RV injury in ARDS, which better align with known RV pathophysiology and outcomes than current RVD definitions: one is compensated RV dilation, and the other is decompensated RV failure with impaired systolic function and cardiac output. They also identify a hyperdynamic left ventricular function, high-cardiac-output subphenotype, which not only overlaps with cardiovascular subgroups in sepsis but also correlates with the hyper-inflammatory subphenotype and indirect subgroup of ARDS. Whether identification of these cardiovascular subphenotypes can improve our understanding of the pathophysiology in shock in ARDS and lead to personalised haemodynamic therapy requires validation in prospective, multi-centre cohorts with protocolised echocardiography, respiratory and biomarker measurement.

## Data Availability

Not applicable.
